# Chronic Inhibition of PDE5 Limits Pro-Inflammatory Monocyte-Macrophage Polarization in Streptozotocin-Induced Diabetic Mice

**DOI:** 10.1371/journal.pone.0126580

**Published:** 2015-05-11

**Authors:** Mary Anna Venneri, Elisa Giannetta, Giuseppe Panio, Rita De Gaetano, Daniele Gianfrilli, Riccardo Pofi, Silvia Masciarelli, Francesco Fazi, Manuela Pellegrini, Andrea Lenzi, Fabio Naro, Andrea M. Isidori

**Affiliations:** 1 Department of Experimental Medicine, Sapienza University of Rome, Rome, Italy; 2 Department of Anatomical, Histological, Forensic and Orthopedic Sciences, Sapienza University, Rome, Italy; 3 Department of Medicine and Health Sciences, University of Molise, Campobasso, Italy; Second University of Naples, ITALY

## Abstract

Diabetes mellitus is characterized by changes in endothelial cells that alter monocyte recruitment, increase classic (M1-type) tissue macrophage infiltration and lead to self-sustained inflammation. Our and other groups recently showed that chronic inhibition of phosphodiesterase-5 (PDE5i) affects circulating cytokine levels in patients with diabetes; whether PDE5i also affects circulating monocytes and tissue inflammatory cell infiltration remains to be established. Using murine streptozotocin (STZ)-induced diabetes and in human vitro cell-cell adhesion models we show that chronic hyperglycemia induces changes in myeloid and endothelial cells that alter monocyte recruitment and lead to self-sustained inflammation. Continuous PDE5i with sildenafil (SILD) expanded tissue anti-inflammatory TIE2-expressing monocytes (TEMs), which are known to limit inflammation and promote tissue repair. Specifically, SILD: 1) normalizes the frequency of circulating pro-inflammatory monocytes triggered by hyperglycemia (53.7 ± 7.9% of CD11b^+^Gr-1^+^ cells in STZ vs. 30.4 ± 8.3% in STZ+SILD and 27.1 ± 1.6% in CTRL, P<0.01); 2) prevents STZ-induced tissue inflammatory infiltration (4-fold increase in F4/80+ macrophages in diabetic vs. control mice) by increasing renal and heart anti-inflammatory TEMs (30.9 ± 3.6% in STZ+SILD vs. 6.9 ± 2.7% in STZ, P <0.01, and 11.6 ± 2.9% in CTRL mice); 3) reduces vascular inflammatory proteins (iNOS, COX2, VCAM-1) promoting tissue protection; 4) lowers monocyte adhesion to human endothelial cells in vitro through the TIE2 receptor. All these changes occurred independently from changes of glycemic status. In summary, we demonstrate that circulating renal and cardiac TEMs are defective in chronic hyperglycemia and that SILD normalizes their levels by facilitating the shift from classic (M1-like) to alternative (M2-like)/TEM macrophage polarization. Restoration of tissue TEMs with PDE5i could represent an additional pharmacological tool to prevent end-organ diabetic complications.

## Introduction

Cardiovascular and renal complications account for the majority of deaths of subjects with diabetes mellitus. Hyperglycemia and oxidative stress are among the main triggers for the development of microvascular complications in both type 1 and type 2 diabetes, as they determine a low-grade chronic inflammatory state that damages the endothelium [1–5]. The low proliferative potential of terminally differentiated endothelial cells is balanced by the contribution of hematopoietic proangiogenic cells that are recruited for tissue repair in various physiologic and pathologic conditions [6–11]. However, hyperglycemia affects both endothelial [12] and immune cell function [13, 14]. Chronic pro-inflammatory M1-type macrophage infiltration has been reported to sustain tissue derangement and insulin resistance [13, 15, 16], linking diabetes to vascular disease [17].

Given that diabetes is largely considered a pro-inflammatory condition, it is surprising that the role of anti-inflammatory cells [18] has rarely been investigated. A current theory postulates that tissue injury in diabetes is worsened by impaired control of the inflammatory response [19], where the final steps (remodeling and repair following injury and inflammation) are defective.

Recent studies showed, that macrophages may display an “alternatively activated” phenotype (“M2-like” as opposed to the “pro-inflammatory M1”), which enhances debris scavenging, angiogenesis and tissue remodeling [20]. Among the markers that characterize this phenotype, the angiopoietin receptor (TIE2) has received particular attention because it favors the association of M2-like macrophages with blood vessels and regulates their ability to induce blood vessel formation in both physiologic and pathologic conditions [7, 8, 21–23]. Importantly, the role of TEMs in the pathogenesis of vascular and tissue inflammation in diabetes has never been previously investigated.

A number of emerging clinical and experimental reports suggest that continuous PDE5 inhibition is associated with cardioprotection, neuroprotection and wound healing [24–28]. In type 2 diabetes patients, we have shown that chronic treatment with sildenafil, a PDE5 inhibitor (PDE5i), is associated with cardioprotection and reduced levels of circulating inflammatory cytokines [29–31]. A promising role for PDE5i in the modulation of inflammatory processes has also been reported in ischemia-reperfusion injury in the heart [24] and in renal damage [32]. Although some of these studies reported improved circulating cytokine profiles and reduced oxidative stress after PDE5i administration, its effects on the composition of the macrophages involved in tissue infiltration remain unclear.

The aim of this study is to investigate if PDE5i might mitigate the M1-type macrophage tissue infiltration induced by hyperglycemia by reducing vascular inflammation through specific modulation of TIE2 expressing monocytes.

## Materials and Methods

### Animal model

Diabetes was induced in 12-week-old male CD1 mice (average body weight 25 ± 15 g) using a single high-dose intraperitoneal injection (i.p.) of Streptozotocin (STZ: 150 mg/kg, Sigma Aldrich) dissolved in saline buffer. After 3 days Sildenafil (1.6 mg/kg, SILD: Viagra, Pfizer in saline 0.3% aqueous solution of DMSO) was administered by i.p. daily, for 3 weeks or, in survival analysis, for 6 weeks. Appropriate vehicle controls (saline or saline 0.3% DMSO) were performed for each setting. Both housing and care of laboratory animals were in accordance with Italian law (D.L. 2010/63EU), and the study was approved by the Sapienza University’s Animal Research Ethics Committee.

### Experimental design

Mice were randomly assigned to 4 groups: CTRL (receiving saline buffer and 3 or 6-weeks DMSO vehicle, n = 14 mice: 7 mice for experimental protocols (Ep) and 7 mice for survival (Sv)), STZ (receiving STZ and 3 or 6 weeks DMSO vehicle, n = 14 mice: 7 mice for Ep and 7 mice for Sv), STZ + SILD (receiving STZ and 3 or 6 weeks SILD treatment, n = 14: 7 mice for Ep and 7 mice for Sv) and SILD (receiving saline buffer and 3 or 6 weeks SILD treatment, n = 7 mice: 3 mice for Ep and 4 mice for Sv). Short term observation (3-week) was used for tissue macrophage infiltration/recruitment analysis and mid-term (6-weeks) for the survival analysis. Mouse sample size was calculated assuming relevant a standardized mean difference of 2 in the percentage of resident TEMs with a 5% significance level and 90% power for independent two-sided tests [33].

All STZ-induced animals were supplied with 10% sucrose water for 72 h after STZ injection to counteract post-injection hypoglycemia. Housing of one mouse per cage allowed individual measurement of food and water intakes. A drop of tail blood after 3h fasting was used to monitor glucose concentrations by MediSense Precision Plus kit (Abbott Diagnostics, Melbourne, Victoria, Australia). Survival studies mice were inspected daily for signs of pain or distress, including changes in respiration, appetite, urine output, excessive thirst, dehydration, activity (for example, lethargy or hyperactivity), weight loss exceeding 10% of the initial value, unkempt appearance, abnormal posture, and twitching or trembling. The measurement of body weight and observation of body condition (for example, thin, normal, overweight) acted as a surrogate marker of appetite. The measurement of skin turgor and observation of cage bedding for urine output acted as a surrogate marker of thirst. These complications were documented, and mice received routine medical management appropriate to presenting symptoms (for example, warming pads for hypothermia, warm physiologic saline for dehydration, dextrose or insulin for metabolic correction, analgesics for management of pain or distress). The follow-up period was kept at 45 d after STZ injection. No animals were found dead. Mice that manifested complications (7 STZ and 3 STZ+SILD) and with a body weight<30% of original body were euthanized promptly with an overdose of carbon dioxide and are plotted in Kaplan-Meier Survival analysis. The criteria for euthanasia of mice were according to Association for Assessment and Accreditation of Laboratory Animal Care of Laboratory Animal Care guidelines.

### Histological and immunofluorescence studies

Tissue specimens were obtained from surgical resections, embedded in OCT compound and snap-frozen. Ten-micrometer sections were fixed in 4% paraformaldehyde and immunostained. For histological examination specimens were processed with hematoxylin-eosin (H&E) reviewed by an experienced pathologist and scored for glomerular and tubulointerstitial injury, as described by Nangaku [34], and for cardiac hypertrophy. For kidney morphological analysis, semi-quantitative scores (0–4) for glomerular and tubule-interstitial injury were assigned in a blinded manner using 15 randomly selected fields of cortex per cross section. Briefly, each single section was graded from 0 to 4, where 0 represents no lesion and 1, 2, 3 and 4 represent mesangial matrix expansion or sclerosis involving ≤25, 25–50, 50–75 and >75% of the glomerular tuft area. Tubulointerstitial injury was graded (0–4+) on the basis of the percentage of tubular cellularity, basement membrane thickening, cell infiltration, tubular dilation, atrophy, sloughing or interstitial widening as follows: 0, no change; 1, <25% tubulointerstitial injury; 2, 25–50% injury; 3, 50–75% injury; and 4, 75–100% injury. The average score was then calculated. For heart analysis: Sections were stained with hematoxylin–eosin and scanned, and the images of surfaces of ventricular cross-sections were measured with the help of Image-J software (National Institutes of Health, Bethesda, MD, USA; http://www.nih). To measure myocyte size, the surface of 50 cells was manually assessed, in at least seven photographs and calculated in μm^2^ with the support of J Image software.

For immunofluorescence staining, frozen sections were pre-blocked with serum and incubated with the following antibodies: 1) rat anti-TIE2, anti F4/80 and anti-CD31 (all from eBioscience), followed by donkey anti–rat Cy3 and FITC–conjugated antibodies (Jackson Immunology), 2) rabbit anti-iNOS (from Cell Signaling) and goat anti-MRC-1 (from R&D System) followed by donkey anti–rabbit and donkey anti–goat Cy3–conjugated antibodies (Jackson Immunology). Cell nuclei were labeled by TO-PRO-3 (Molecular Probes). Image analysis was performed with Leica confocal microscopy (TCS-SP2) and by Image-J software (NIH, Bethesda, USA). Capillary density was quantified from at least five high-power fields (×40) per slide, two slides per animal (3 animals per group). Sections were randomly selected, and analyzed in blind.

### Flow cytometry

For tissue specimens, mice were transcardially perfused with phosphate-buffered saline (PBS). Organs were reduced to single-cell suspensions by 2 digestions with collagenase type 2 (Sigma) and collagenase/dispase (Roche). Preparations were passed through a 40-μm cell strainer (Becton Dickenson) and washed. The resulting single cells were collected, blocked in 5% FBS in PBS, centrifuged and then stained with the following antibodies: rat anti F4/80-APC (eBioscience), rat anti TIE2-PE (eBioscience) and rat anti-CD11b-PE-Cy7C conjugated. Cells were labeled using 7-amino-actinomyocin D before analysis to detect necrotic cells.

Blood collected from each animal before and 3 days after STZ/CTRL treatment and then at weekly intervals during SILD/vehicle treatment was anti-coagulated with EDTA at room temperature. Ammonium Chloride solution was used for 10 minutes at RT to allow red blood cell lysis. Cells were stained with rat anti-CD11b conjugated in PE-Cy7 (eBioscience) and rat anti-Ly-6G conjugated in APC (eBioscience) and were then centrifuged at 1200 RPM for 10 min at 4°C. Typical antibody-specific dilutions were used according to supplier datasheet. All samples were collected by a CyAn ADP cytometer (DAKO). Biexponential analysis was performed using Summit V4.3 software.

### Protein analysis

Kidney and heart lysates were made by homogenizing pulverized, freeze-clamped tissue in lysis buffer (150 mM NaCl, 1% v/v NP-40, 0.5% w/v sodium deoxycholate, 0.1% w/v SDS, 50 mM Tris-HCl pH 7.6) with added protease inhibitor (Sigma). Samples were run on Novex 4–12% Tris-glycine gels (Invitrogen), transferred to PVDF membranes and incubated with anti β-Myosin, anti-GAPDH and anti-VCAM (Santa Cruz Biotechnology), anti-COX-2, iNOS and Nf-kB (Cell Signaling) antibodies. Densitometry was performed with AIDA 2.2 and Image J software.

### mRNA quantitative analysis

Total RNA was isolated from heart tissue using TRI Reagent based on the company protocol (Sigma). RNA concentration was determined by spectrophotometry at 260 nm. Reverse transcription was performed using High Capacity RNA-to-cDNA kit (Applied Biosystems) in a total volume of 20 μl incubated for 1 h at 37°C, and stopped by heating for 5 min at 95°C.

Quantitative PCR assay for ANP was performed on an ABI 7500 Fast Real-Time PCR System using TaqMan Fast Universal PCR master mixture (Applied Biosystems) and TaqMan Gene Expression Assay ANP (NNPPA Mm01255747_ g1). The amplicon size was 85. After 2 min at 50°C to allow AmpErase uracil-N-glycosilase (UNG) to destroy potential contaminant PCR products and 10min at 95 grade to denaturate UNG and activate Taq polymerase, the amplification was carried out over 45 cycles of 15 sec at 95°C and 1 min at 60°C. All pcr reactions were performed in triplicate for this target gene and the internal control. Relative gene expressions were presented with the -2ΔΔCt method.

### Human in vitro endothelial assay

Monocyte adhesion assays were performed on confluent HUVEC (ATCC CRL-1730) cells grown on 6-well plates. HUVEC monolayers were treated with low (5 mM) or high (30 mM) glucose in EGM-2 medium for 12 hours, in the presence or absence of SILD (2 μmol), TIE2-Fc (2 μg/mL); THP-1 (ATCC TIB-202) cells were then added to the HUVEC monolayers for another hour. After incubation, unbound cells were washed off to visualize the bound monocytes. Digital images of nine random fields per well were counted at 10x power to determine the mean number of adherent monocytes. Nikon Eclipse TS100 Microscopy was used to collected images.

### Statistical analysis

Data were analyzed by Prism 6 software. Results are expressed as mean ± SD. Quantitative data were analyzed and compared using Student’s t test for comparison of two groups. All P values are two-tailed, and values less than 0.05 indicate statistical significance.

## Results

### Effects of SILD on metabolism and survival of STZ-induced diabetic mice

**Fig 1:**
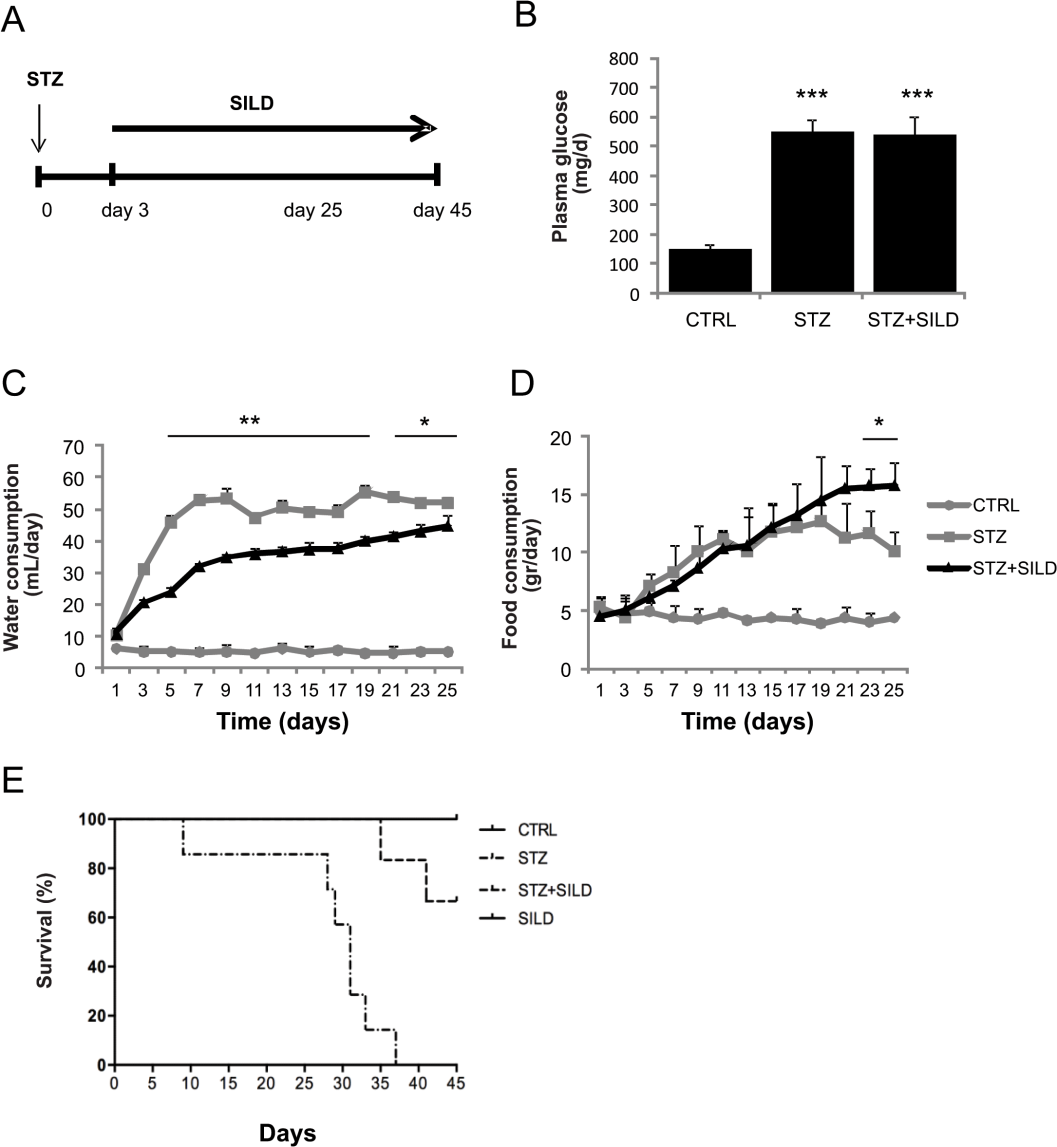
Metabolic characteristics and survival of treated mice. (A) Schematic representation of the experimental design. (B) Plasma glucose levels in diabetic (STZ and STZ+SILD) vs. non-diabetic (CTRL) mice. Results are expressed as mean ± SD (n = 7 in each group). (C) Changes in water consumption in STZ and STZ+SILD vs. CTRL mice. Results are expressed as mean ± SD (n = 4 in each group). (D) Changes in food consumption index in STZ and STZ+SILD vs. CTRL mice. Results are expressed as mean ± SD (n = 4 in each group). (E) Kaplan-Meier survival curve of CTRL, SILD, STZ and STZ+SILD mice.

Diabetes was induced in CD1 mice by a single dose (150 mg/kg) of STZ and achieved in 95% of mice by day 5, according to serum glucose levels (Fig 1B); 1.6 mg/kg of SILD was administered daily from day 3 to 45 after SZT injection (Fig 1A). Blood glucose levels were significantly higher in STZ-diabetic mice than in CTRL mice (434.3 ± 35.1 vs. 113.0 ± 2.0 mg/dl; p<0.001, Fig 1B), and SILD did not affect glucose levels in the double-treated STZ+SILD mice (433.9 ± 16.7 mg/dl), suggesting that SILD had no effect on hyperglycemia (Fig 1B).

Water intake increased gradually from day 3 after STZ, peaked at day 9 and remained stable thereafter (Fig 1C). Diabetic mice receiving sildenafil (STZ+SILD) showed a slightly lower water intake than vehicle-treated diabetic mice (STZ) (*P<0.05 and **P<0.01) and a slightly higher food intake at the end of observation only (Fig 1C and 1D). In non-STZ treated mice, there were no changes in food consumption, water intake or blood glucose after vehicle or SILD treatment. Kaplan-Meier survival curves for treated animals are reported in Fig 1E: 100% of STZ animals died within 45 days of observation compared to only 28% of STZ+SILD treated mice (Fig 1E, STZ vs. STZ+SILD P<0.01). Since there were no differences in glycemic status between the groups, we hypothesized that PDE5i protected against diabetic end-organ complications though an independent mechanism.

### SILD restores alterations of CD11b+Gr-1+ and CD11b+Gr-1 circulating myeloid cells in diabetic mice

**Fig 2:**
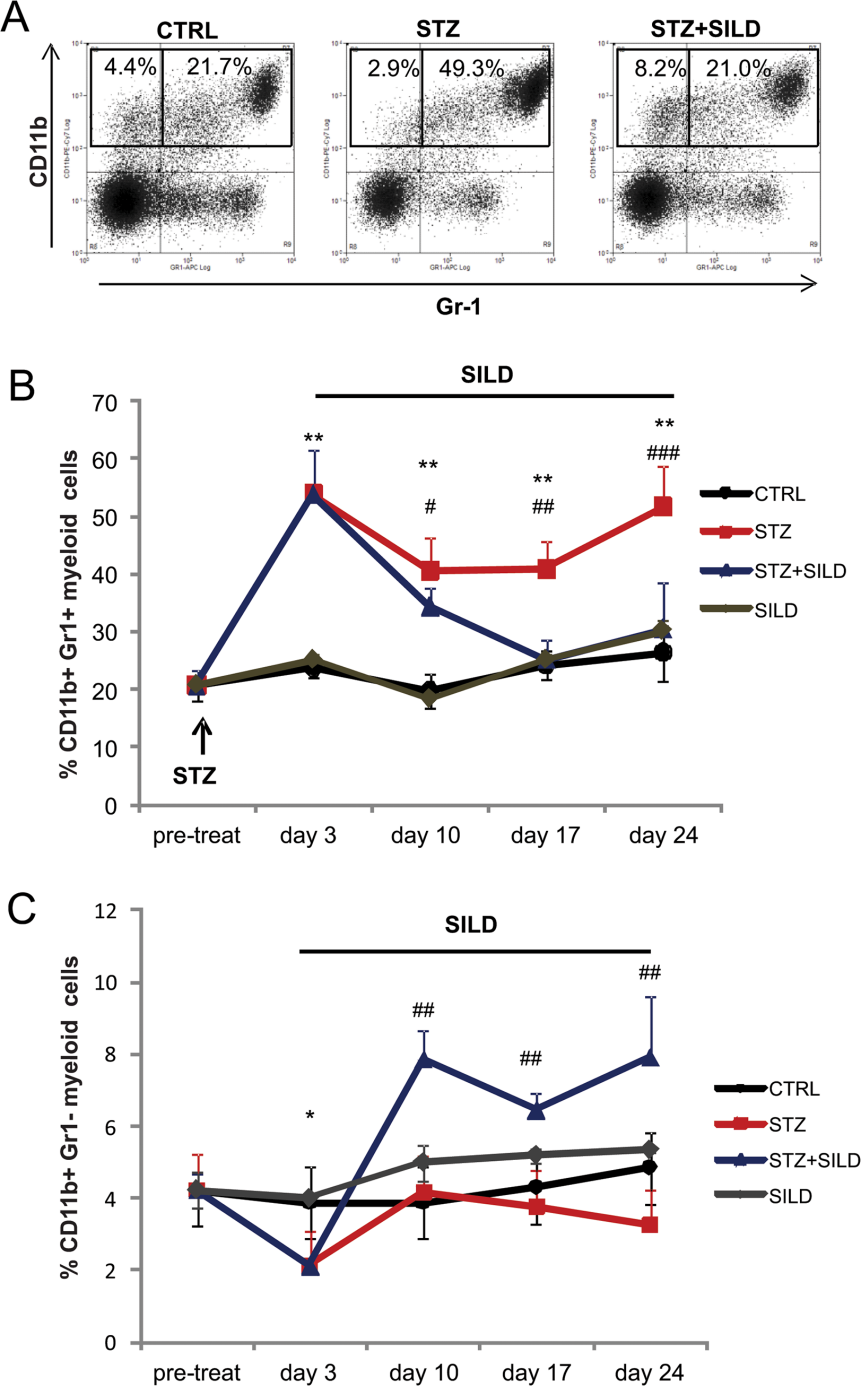
SILD restores circulating CD11b^+^Gr1^+^ and CD11b^+^Gr1^-^ myeloid cells. (A) Immunophenotype of circulating myeloid cells from one of several independent experiments is shown for animals from the CTRL, STZ and STZ+SILD groups; blood was drawn at day 24. Monocytes identified on the basis of expression of CD11b and GR-1 marked cells are gated (open black line in the plots). (B) Percentages of CD11b^**+**^ and GR-1^**+**^ marked cells in CTRL, SILD, STZ and STZ+SILD mice before STZ treatment and after 3, 10, 17 and 21 days in each of the four treatment groups. Results are expressed as mean ± SD (n = 7 in each group), ^**^ P <0.01 STZ vs CTRL, ^**#**^ P <0.05, ^**##**^ P <0.01, ^**###**^ P <0.001 STZ+SILD vs STZ). (C) Percentages of CD11b^**+**^ and GR1^-^ marked cells in CTRL, SILD, STZ and STZ+SILD mice before STZ treatment and after 3, 10, 17 and 21 days in each group. Results are expressed as mean ± SD (n = 7 in each group, ^*^ P <0.05 STZ vs CTRL, ^**##**^ P <0.01 STZ+SILD vs STZ).

To investigate whether SILD modifies the inflammatory response to high-dose STZ-induced hyperglycemia, we analyzed CD11b^+^GR1^+^ and CD11b^+^GR1^-^ myeloid cells, that are involved in tissue remodeling. In STZ mice we found a significant change in circulating myeloid cells, starting three days after STZ injection and stable thereafter, with increased expression of CD11b^+^Gr-1^+^ (53.7 ± 7.9 STZ, 27.1 ± 1.6 CTRL, P<0.01; Fig 2A and 2B) and reduced CD11b^+^Gr-1 cells (2.1 ± 0.6 STZ, 3.7 ± 1.2 CTRL, P<0.05; Fig 2A–2C). In diabetic mice receiving SILD, CD11b^+^Gr-1^+^ returned to the baseline level (30.4 ± 8.3, P<0.01) after 3 weeks of treatment, while CD11b^+^Gr-1 (7.9 ± 1.3, P<0.01) remained stably higher than in STZ-only treated mice (Fig 2C).

This suggests that SILD affects circulating myeloid subpopulations in diabetes by lowering CD11b^+^GR1^+^ levels to those of non-diabetic mice and increasing the relative percentage of CD11b^+^GR1^-^ cells.

### SILD mitigates diabetic renal macrophages infiltration

**Fig 3:**
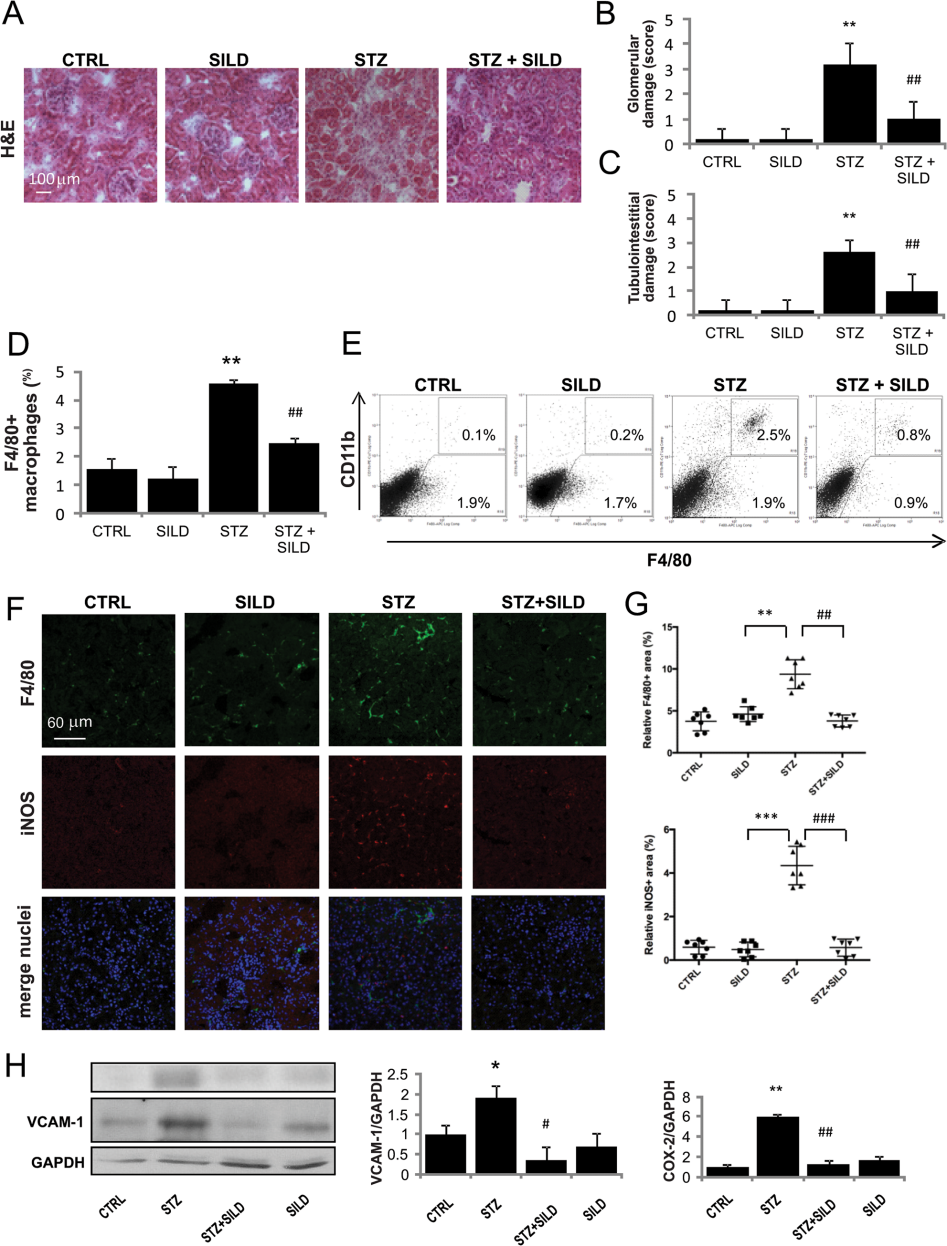
Effect of SILD on STZ-diabetic kidney: modulation of macrophage infiltration. (A) Representative photomicrographs of H&E-stained kidneys (×200 magniﬁcation) on harvesting show normal structure in the CTRL and SILD, mild mesangial expansion and tubular damage in the STZ, and normal structure in the STZ+SILD group (bar represents 100 μm).(B) and (C) Glomerular and tubulointerstitial damage index scores are shown for each group. Data are expressed as mean ± SD. ^**^P <0.01 STZ vs CTRL; ^**##**^P <0.01 vs STZ (Student’s t-test).(D) Histograms indicate percentage of F4/80^**+**^ cells. Results are expressed as mean ± SD (n = 4 in each group) ^**^P <0.01 STZ vs CTRL; ^**##**^P <0.01 STZ+SILD vs STZ (Student’s t-test).(E) Immunophenotype of infiltrating macrophages in kidneys from representative CTRL, SILD, STZ and STZ+SILD mice, performed to assess expression of F4/80 and CD11b. Percentage of F4/80^**+**^ cells is augmented in STZ mice. One experiment representative of several independent experiments is shown.(F) Confocal analysis for F4/80 (green) and iNOS (red) shows abundant interstitial inflammatory macrophages (iNOS^**+**^) in renal sections from STZ mice, that were significantly decreased in STZ+SILD mice. (G) Relative F4/80^**+**^ and iNOS^**+**^ area quantification. Each dot represents one slide in different kidney groups.(H) Quantification of VCAM-1 and COX-2 to GAPDH expression detected by Western blotting reveals vascular inflammation in kidneys from STZ mice and less damage in kidneys from STZ+SILD mice.

To evaluate whether the observed changes in circulating monocytes were mirrored by changes in tissue inflammatory infiltration, we investigated highly vascular organs susceptible to metabolic derangement. STZ-mice kidneys revealed extensive glomerular damage, mesangial expansion, tubular dilatations and atrophy, all of which were less frequently observed in STZ+SILD animals (Fig 3A,3B and 3C).

The number of macrophages in kidneys made into single-cell suspensions and assessed by F4/80^+^ macrophages marker by flow cytometry, was drastically increased in diabetic mice (Fig 3D). Analysis revealed a 4-fold increase in F4/80^+^ macrophages in diabetic vs. control mice (4.6 ± 0.2 STZ, 1.2 ± 0.8 CTRL, P <0.01), while SILD prevented macrophage infiltration (2.5 ± 0.2, P <0.01 vs. STZ). Interestingly, we found that a greater number of F4/80^+^ macrophages of STZ mice co-expressed CD11b, an integrin involved in monocyte-to-endothelium adhesion, indicating that recent cell extravasation was more likely than local macrophage expansion (Fig 3E). In addition, we noted that accumulation of the F4/80^+^ population in the glomeruli and tubulointerstitium of STZ mice, that was correlated with increased expression of M1-type genes such as iNOS, was promptly counteracted by SILD treatment (Fig 3F and 3G).

In diabetes, macrophages are recruited to sites of vascular injury and roll along the vascular endothelium where VCAM-1 plays a crucial role in priming vascular inflammation [35]. Increased renal cyclooxygenase-2 (COX-2) activity has been linked to the hyper-infiltration of diabetes [36]. As expected, levels of the vascular inflammatory proteins VCAM-1 and COX-2 were very low in controls, increased dramatically in STZ and dropped in STZ+SILD kidneys, suggesting that PDE5i limits vascular inflammation (Fig 3H). These data reveal significant modulation of cell recruitment and expression of the genes involved in inflammatory vascular extravasation.

### SILD increases renal resident anti-inflammatory TEMs

**Fig 4:**
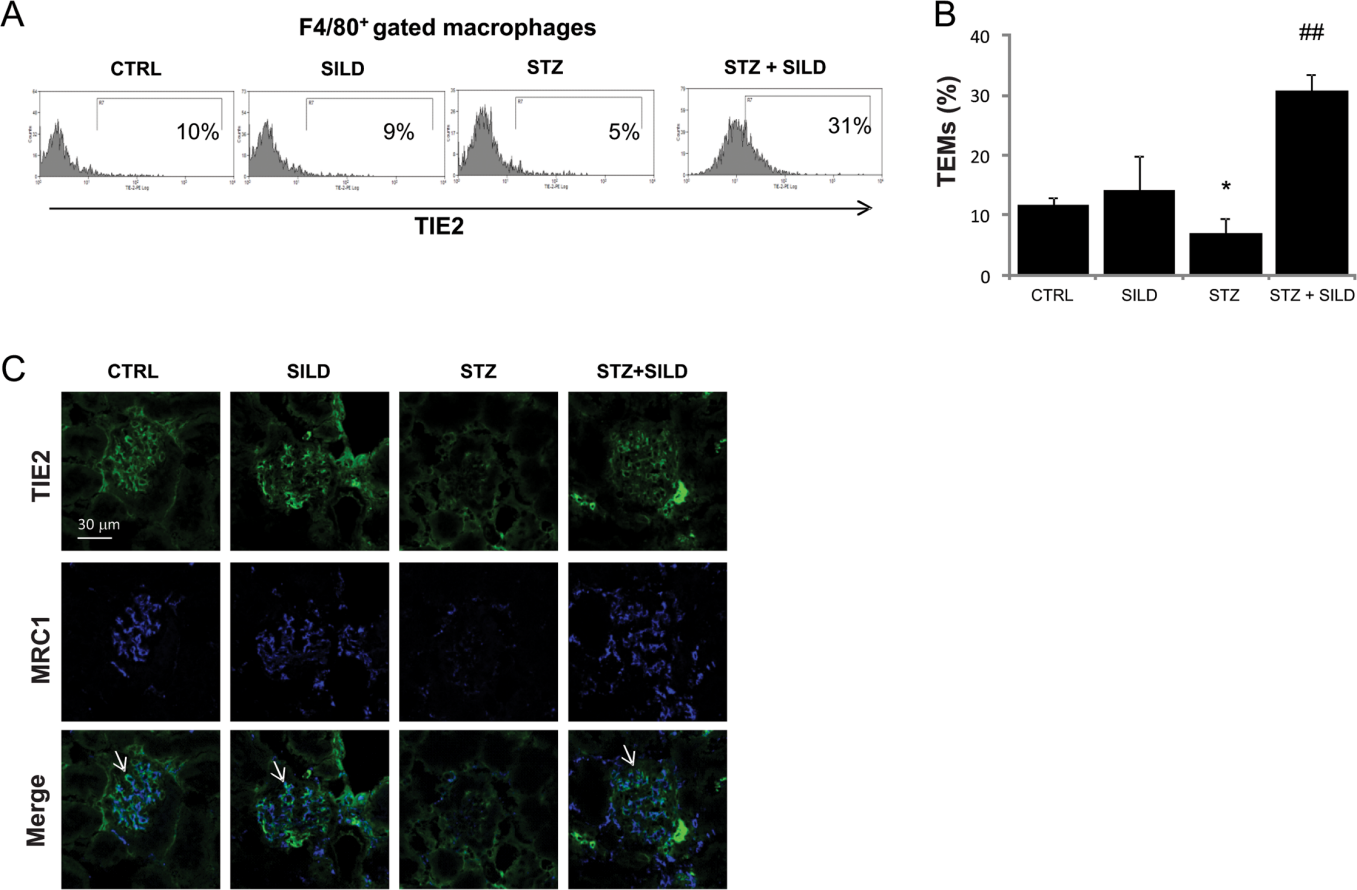
SILD increases renal expression of TEMs pro-angiogenic macrophages. (A) Representative of at least 4 flow cytometry analyses of macrophage infiltration in kidney from CTRL, SILD, STZ and STZ+SILD mice, showing TIE2 expression in a subset of monocytes (filled line in the histogram plots).(B) Histograms indicate percentage of TIE2^**+**^ in the F4/80^**+**^ macrophages population. Expression of F4/80^**+**^TIE2^**+**^ cells decreased in STZ compared to CTRL mice and increased in the STZ+SILD compared to STZ mice. Results are expressed as mean ± SD (n = 4 in each group); ^*^P <0.01 STZ vs CTRL; ^**##**^P <0.01 STZ+SILD vs STZ (Student’s t-test).(C) Confocal analysis for kidney TEMs from representative CTRL, SILD, STZ and STZ+SILD mice, performed for TIE2 (green) and MRC1 (blu). TIE2^**+**^MRC1^**+**^ macrophages were lower in STZ than in CTRL and higher in SILD and STZ+SILD groups (bar represents 30μm).

Macrophages exhibit a range of phenotypes that are associated with distinct functions, a phenomenon that has been described as “polarization”. To test whether the improved tissue protection associated with SILD treatment was achieved through an alternative polarization of tissue macrophages, we analyzed the expression of TIE2, a marker for anti-inflammatory pro-angiogenic M2 macrophages. In STZ mice, we observed a lower percentage of TEMs compared with CTRL (6.9 ± 2.7 STZ, 11.6 ± 2.9 CTRL, P <0.05). Surprisingly, the number of TEMs in the STZ+SILD mice was significantly higher than in both the STZ and the CTRL mice (30.9 ± 3.6, P <0.01 vs. STZ; Fig 4A and 4B).

TIE2 expression in renal parenchyma was pronounced in fenestrated glomerular capillaries in CTRL, SILD and STZ+SILD treated animals, but reduced in STZ-only animals (Fig 4C). A proportion of TIE2^+^ cells co-localized with mannose receptor-1 (MRC1), a TEM marker, intermixed by TIE2^+^MRC1^-^ endothelial cells. While TEM cells were observed in CTRL, SILD and STZ+SILD mice, they were nearly absent in the glomeruli of STZ mice (Fig 4C). Taken together, these findings indicate that SILD drives the response to acute hyperglycemic renal injury from a classic pro-inflammatory M1 type toward the alternative pro-angiogenic TEM phenotype.

### SILD protects against cardiac macrophage infiltration

**Fig 5:**
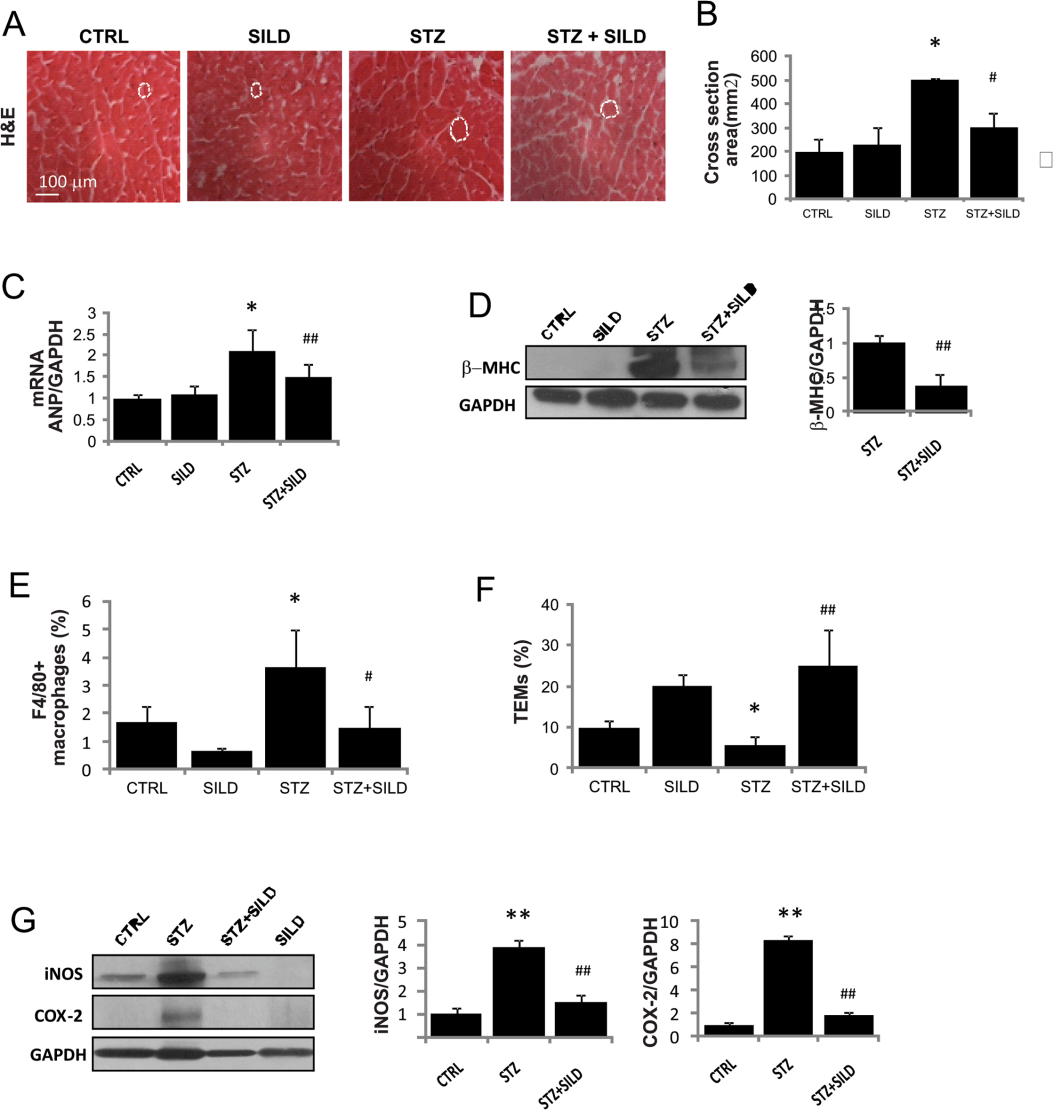
Effect of SILD on cardiac hypertrophy and macrophage infiltration. (A) Representative photomicrographs of H&E-stained heart from CTRL, SILD, STZ and STZ+SILD mice on harvesting (bar represents 100μm).(B) Histogram shows heart cross-sectional area. Results are expressed as mean ± SD (n = 4 in each group). ^*^P <0.05 STZ vs CTRL; ^#^P <0.05 STZ+SILD vs STZ (Student’s t-test).(C) Histogram shows mRNA quantitative analysis ANP. Results are expressed as mean ± SD (n = 3 in each group). ^*^P <0.05 STZ vs CTRL; ^##^P <0.01 STZ+SILD vs STZ (Student’s t-test).(D) β-MHC and GAPDH expression detected by Western blotting. Histogram shows activation of hypertrophic protein in STZ mice that was reduced in STZ+SILD mice. Results are expressed as mean ± SD (n = 3 in each group) ^##^P <0.01 STZ+SILD vs STZ (Student’s t-test).(E) Histogram illustrates percentage of infiltrating F4/80^+^ cells in the heart from CTRL, SILD, STZ and STZ+SILD mice. Results are expressed as mean ± SD (n = 4 in each group). ^*^P <0.05 STZ vs CTRL; ^#^P <0.0 STZ+SILD vs STZ (Student’s t-test).(F) Histograms indicates percentage of F4/80^+^TIE2^+^ cells. Expression of F4/80^+^TIE2^+^ cells was decreased in STZ compared with CTRL mice and increased in STZ+SILD compared with STZ mice. Results are expressed as mean ± SD (n = 4 in each group), ^*^P <0.05 STZ vs CTRL; ^##^P <0.01 STZ+SILD vs STZ (Student’s t-test).(G) iNOS and COX-2 expression detected by Western blotting. Histogram shows induction of inflammatory proteins in STZ mice that was reduced in STZ+SILD mice. Results are expressed as mean ± SD (n = 3 in each group). ^**^P <0.01 STZ vs CTRL, ^##^P <0.01 STZ+SILD vs STZ (Student’s t-test).

Several studies report that high-dose STZ-induced diabetes is associated with cardiac hypertrophy and fibrosis [37]. This is supported by our finding of increased heart size (heart weight/tibial length) (Table 1) and cardiomyocyte cross-sectional areas in diabetic hearts, partially reversed by SILD treatment (Fig 5A and 5B). Parameters of ventricular hypertrophy such as ANP and beta-myosin were increased by STZ and lowered by SILD (Fig 5C and 5D).

**Table 1:** Mice group characteristics after 3-week sildenafil treatment.

	CTRL (*n* = 7)	STZ (*n* = 7)	STZ+ SILD (*n* = 7)	SILD (n = 3)
Body weight before treatment (g)	34±5.5	36±2.7	37±7.2	35± 5.5
Body weight following treatment (g)	36±5.1	30±5.7	33±4.0	36± 4.5
Heart weight (mg)	148.4±42.0	170.6±41.1^aa^	157.1±46.2^b^	148.2± 42.0
Tibial lenght (cm)	1.9±0.1	1.9±0.1	2.0±0.2	1.9± 0.1
Heart weight/tibial lenght	78.1±23.1	89.8±19.5^a^	78.5±19.7^b^	78.0± 20.5

Values are presented as mean ± SD, ^a^P <0.05 and ^aa^P <0.01, STZ treated versus normal and ^b^P<0.05, STZ+SILD treated versus STZ.

Heart tissue suspensions were analyzed by flow cytometry and we found a 2-fold increase in F4/80^+^ cells in diabetic mice compared with controls (3.7 ± 1.5 vs. 1.7 ± 0.3, P <0.05), blunted by SILD (1.5 ± 0.4, P <0.05 vs. STZ; Fig 5E). There was a decrease in TEMs in the STZ mice compared with CTRL (5.8 ± 0.9, 9.7 ± 3.6, P <0.05) that was reverted in STZ+SILD (25.1 ± 8.5, P <0.01; Fig 5F). As expected, COX-2 and iNOS protein levels were very low in controls, dramatically increased in STZ and reduced in STZ+SILD hearts, suggesting that PDE5i limits inflammatory processes (Fig 5G).

Overall, these findings suggest that SILD reduces cardiac microvascular inflammation by enhancing TEM infiltration.

### High glucose-induced vascular inflammation is inhibited *in vitro* by SILD via the TIE2 receptor

**Fig 6:**
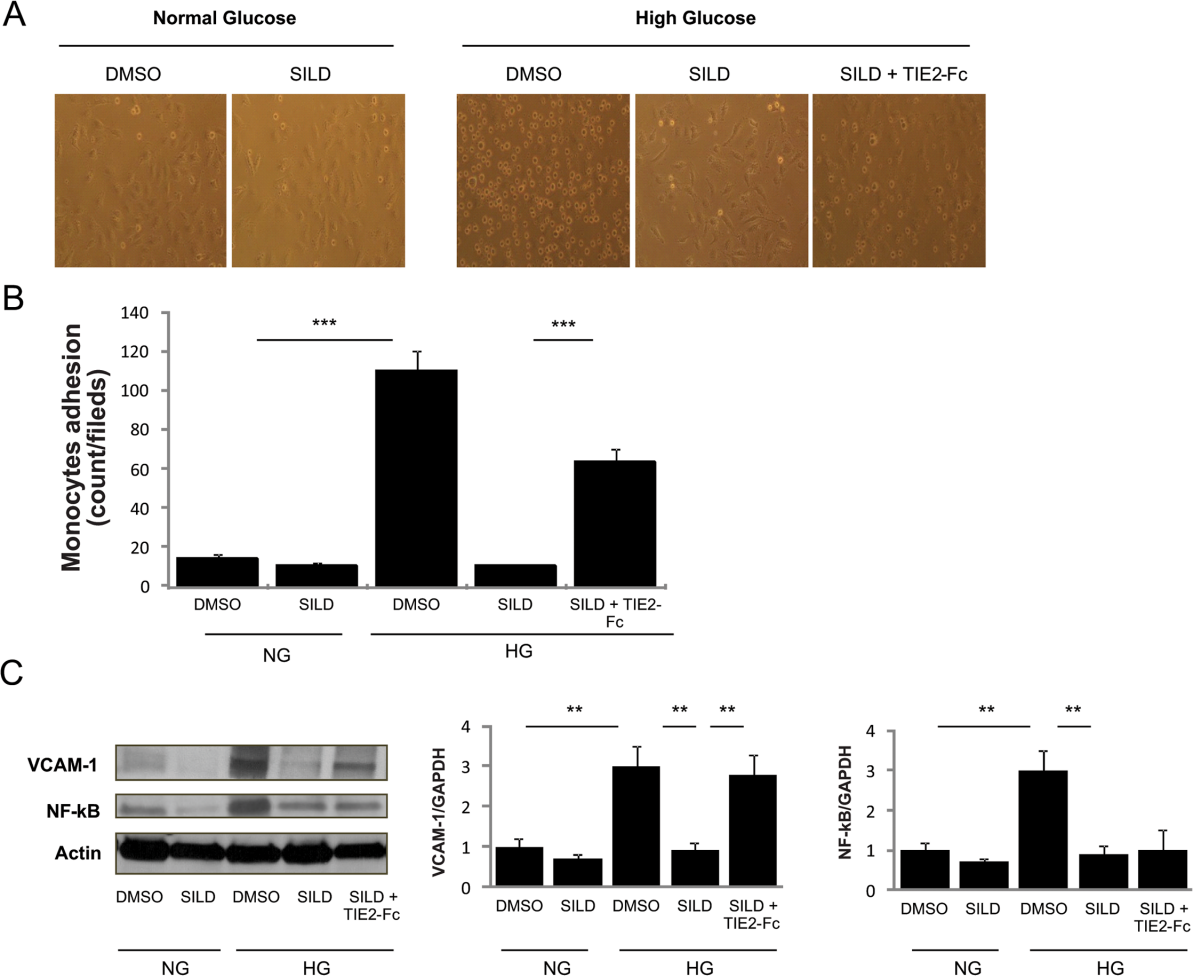
SILD inhibits high-glucose-induced adhesion via TIE2 receptor. (A) THP1 cell adhesion to HUVEC. Note that there are more adherent monocyte cells in high-glucose than in normal condition medium. SILD inhibits high-glucose-induced adhesion and co-treatment with TIE2-Fc cancelled this effect (n = 3/treatment group). (B) Quantification of monocyte adhesion to HUVEC. Histogram shows number of adherent cells per field in different experimental groups (n = 3/group). Results are shown as mean ± SD (n = 5 in each group). ^***^ P <0.001. ^**^ P <0.01 (Student’s t-test).(C) VCAM-1, NF-kB and GAPDH expression detected by Western blotting reveals that SILD inhibits vascular inflammation induced by high glucose treated HUVEC, while co-treatment TIE2-Fc cancelled this effect in VCAM-1.

One of the earliest events in the vascular inﬂammation process is adhesion of monocytes to the endothelium, which is followed by their inﬁltration and differentiation into macrophages. To break down the complex mechanism observed *in vivo*, we explored monocyte adhesion of THP1 to human endothelial cells (HUVEC) in the presence of high glucose (HG) *in vitro*. We confirmed previous observations showing that HG promotes monocyte adhesion to HUVEC cells in the presence of high (30 mM) glucose concentrations (P <0.01). Interestingly, monocyte adhesion was restored to baseline levels when SILD was added to the culture (Fig 6A and 6B). We then investigated the involvement of the TIE2 receptor in this setting finding that co-treatment of SILD with soluble TIE2-Fc nullified the effect of SILD. In addition, HG-induced upregulation of VCAM-1 and nuclear factor kappa-light-chain-enhancer of activated B cells (NF-kB) protein were both modulated by SILD; however, only VCAM-1 downregulation by SILD occurred via the TIE2 receptor, as it was abolished by TIE2-Fc (Fig 6C).

To summarize, SILD protects endothelial cells from HG-induced inflammation by inhibiting the expression of VCAM-1 on endothelial cells via TIE2 activation, thus limiting monocyte adhesion and extravasation.

## Discussion

This study identifies a new biological action for PDE5 inhibitors in modulating a specific subset of M2 macrophages that are reduced in STZ-induced diabetic mice, and normalized by SILD with beneficial effects on vascular inflammation and tissue remodeling.

Impaired tissue repair is a common feature of end-organ complications of type-1 and type-2 diabetes. Hyperglycemia and oxidative damage are recognized triggers for endothelial, smooth muscle cell dysfunction and a pro-inflammatory/thrombotic state [3, 38–40]. Monocytes are emerging as potential targets in a number of diabetes-related disorders [14, 18, 41], and their recruitment to different sites is a dynamic process that modifies the pool of resident macrophages [42]. Tissue injuries produce specific changes in this dynamic pool, with the first stage involving enhanced pro-inflammatory M1 cell recruitment. Our data confirm that hyperglycemia itself promotes monocyte adhesion and that STZ-induced diabetes is associated with up-regulation of the molecular machinery necessary for macrophage extravasation. While the majority of studies focus on humoral changes, such as circulating cytokines, more recent studies showed that specific variations in monocyte subsets [18] such as classical human CD14^+^CD16^-^ monocytes have been correlated with poorer outcomes in diabetic patients [41]. Our data confirm that M1 macrophages infiltration is associated with reduced survival and greater tissue damage. Whether this is the result only of aggression from classical monocytes, or of a combination of both injury and hampered tissue repair, remains unclear.

Endothelial cells play a crucial role by releasing multiple inflammatory mediators and expressing various adhesion molecules, including VCAM-1[43]. This membrane protein is necessary to anchor leukocytes to the vessel wall and is an established marker of endothelial dysfunction [35]. Renal COX-2 activity is also increased in diabetes [44] and is linked to hyper-infiltration. Consistently with this, we found increased VCAM-1 and COX-2 protein expression in diabetic kidneys, accompanied by massive F4/80^+^iNOS^+^ M1 macrophage infiltration, and in human endothelial cells exposed to HG, in which monocyte adhesion is enhanced. Interestingly, SILD reduced VCAM-1 expression in mouse tissues and human endothelial cells, lowering monocyte adhesion via TIE2, suggesting that an intact TIE2 receptor is both necessary and sufficient to protect the endothelium.

In addition to the emerging evidence supporting the role of PDE5is as inflammation modulators [24, 28, 32], we characterized, for the first time, the composition of macrophage infiltration and demonstrated a defective recruitment of a specific M2 subset in response to hyperglycemia. The pro-angiogenic tissue macrophages identified here are highly reminiscent of the M2-like pro-angiogenic TEMs that we have described in tumors, developing organs and regenerating tissues [6–8, 21–23]. We found that TEM reduction was associated with specific tissue damage and reduced overall survival of STZ mice. Interestingly, we also found that PDE5 inhibition both restores TEMs and prevent pro-inflammatory circulating monocyte expansion triggered by hyperglycemic stress. It is worth noting that our data reveal a specific hyperglycemia-associated reduction in TEMs rather than a global reduction in the total number of MRC-1^+^ M2 macrophages. The effects of PDE5i thus appear to be specific to a distinct, but relevant, part of the inflammatory process. Long term observation could be of particular interest to address adaptive changes to chronic inflammatory status.

In this respect, previous clinical trials from our group support the use of PDE5i in preventing end-organ complications of diabetes [29, 31, 32]. The improved survival in the current experimental model is consistent with several others reporting tissue protection from PDE5 [25–27, 45, 46].

In this scenario, TEMs could also serve as a new, robust and accessible marker to monitor vascular complications in diabetes. Future work is awaited to provide more information on monocyte function abnormalities as potential targets to improve the diagnosis and treatment of vascular disorders in diabetes.
